# Insights into Grain Milling and Fractionation Practices for Improved Food Sustainability with Emphasis on Wheat and Peas

**DOI:** 10.3390/foods13101532

**Published:** 2024-05-15

**Authors:** El-Sayed M. Abdel-Aal

**Affiliations:** Guelph Research and Development Centre, Agriculture and Agri-Food Canada, Guelph, ON N1G 5C9, Canada; elsayed.abdelaal@agr.gc.ca; Tel.: +1-519-362-1673; Fax: +1-226-217-8181

**Keywords:** cereals, legumes, milling limitations, wheat fractionation, peas fractionation, sustainable milling

## Abstract

Cereal grains and pulses are staple foods worldwide, being the primary supply of energy, protein, and fiber in human diets. The current practice of milling and fractionation yields large quantities of byproducts and waste, which are largely downgraded and end up as animal feeds or fertilizers. This adversely affects food security and the environment, and definitely implies an urgent need for a sustainable grain processing system to rectify the current issues, particularly the management of waste and excessive use of water and energy. The current review intends to discuss the limitations and flaws of the existing practice of grain milling and fractionation, along with potential solutions to make it more sustainable, with an emphasis on wheat and peas as common fractionation crops. This review discusses a proposed sustainable grain processing system for the fractionation of wheat or peas into flour, protein, starch, and value-added components. The proposed system is a hybrid model that combines dry and wet fractionation processes in conjunction with the implementation of three principles, namely, integration, recycling, and upcycling, to improve component separation efficiency and value addition and minimize grain milling waste. The three principles are critical in making grain processing more efficient in terms of the management of waste and resources. Overall, this review provides potential solutions for how to make the grain processing system more sustainable.

## 1. Introduction

Cereals and legumes are staple foods worldwide being the primary supply of energy, protein and fiber in human diets [1]. In addition, they are good sources of micronutrients such as vitamins and minerals as well as health-promoting components including polyphenols, carotenoids and many other bioactive compounds [2,3,4,5,6]. Both food plants are also good candidates for sustainable food production systems and a healthy planet due to their complementary characteristics when they are sown together in a crop rotation system, i.e., they improve soil structure, fertility and microorganisms resulting in better crop yields [7], and an improved ecosystem. Furthermore, plant foods have competitive advantages over animal proteins due to the higher greenhouse gas (GHG) emissions produced from the production of animal-based foods which are approximately twice as that of plant-based foods [8]. Besides, plant foods require lower production cost and energy than animal foods [9]. These attributes signify the importance of cereals and legumes in human nutrition and the health of environment.

Research has shown the role of grains and legumes in the promotion of human health and the prevention of non-communicable diseases, such as diabetes, cardiovascular diseases, and some cancers [10] due to their roles as potent antioxidants [11] and anti-inflammatory substances [12,13]. Interestingly, a combination of cereal grains and pulses (legume dry seeds) in the human diet improves the biological quality of proteins, as they complement one another in terms of their content of limiting amino acids, i.e., cereals boost sulfur amino acids, while pulses increase lysine [14]. Additionally, their combined flavanols have also been found to elicit strong synergistic anti-inflammatory effects and health-enhancing attributes [15]. Surely, the nutritional and health-enhancing attributes of cereals and pulses make them the basis of sustainable and healthful diets for humans.

As the world population is steadily increasing, sustainable food systems are urgently needed more than ever to feed the planet’s inhabitants. The world population is projected to be 8.5 billion in 2030 and to reach 9.7 billion by 2050 and 10.4 billion in 2100 [16]; thus, more foods are needed in the future to feed about 1.2 and 1.9 billion more people in 2050 and 2100, respectively. Alongside the dramatic increase in the world population, over one-third of all food produced (2.5 billion tons) is lost or wasted each year, which contributes to food insecurity and the environmental footprint [17]. These issues definitely require a comprehensive approach with a new vision to develop mitigation strategies capable of promoting food security, economic growth, and sustainable agriculture. Because plant-based foods, including grains, pulses, vegetables, and fruits, are more appropriate for the health of humans and the environment [18], cereals and legumes can be key elements of sustainable food system strategies. The current article is intended to give new insights into the milling and fractionation of grains and pulses in terms of current issues and potential solutions to improve the sustainability of this important food segment.

## 2. Characterization and Fractionation of Grains

Cereal grains, such as wheat, corn, and rice, are commonly referred to as cereals, while legume dry seeds, including beans, peas, and lentils, are known as pulses. Both cereals and pulses are staple foods worldwide; some of them are consumed in their intact forms after cooking, either at home or in a processed form such as canned beans, peas, and corn. Nevertheless, large quantities of cereal grains and pulses are commercially processed into flours, starches, proteins, and other milling fractions for use in food formulations and/or non-food applications. Cereals and pulses possess distinct structures in addition to diverse nutrient and health-enhancing components. Cereals belong to the grass family (*Poaceae or Gramineae*), and the edible grain, kernel, or caryopsis comprises endosperm, germ, and outer layers (bran). For wheat kernel, the proportions of these parts are 81–84%, 2.5–3.6%, and 14–17%, respectively [19]. Pulses belong to the leguminous family (*Leguminosae*), and the seed consists of cotyledons, seed coats (hulls), and embryos [20]. In general, these parts make up approximately 80%, 15%, and 4% of the total seed weight, respectively. Additionally, cereals are monocots, whereas pulses are dicots whose seeds develop differently, especially with respect to the major storage organs [21]. These differences in the structure of cereals and pulses, in addition to the uniqueness of each grain or seed, make their milling and fractionation into botanical parts and/or chemical components distinctively challenging.

Cereal grains and pulses are considered good sources of starch, protein, and fiber with a wide range of characteristics and functionalities. Both food crops are traditionally processed into flour, starch, protein, and fiber. In addition to starch, protein, and fiber, other cereals are rich in oil, like corn, or contain reasonable quantities of β-glucan, such as oats and barley, or arabinoxylan like rye or wheat bran. The latter cereals can also be processed to render oil, β-glucan, or arabinoxylan. In general, dry and wet milling are commonly employed by the grain industry to fractionate grains or pulses into their constituents of protein, starch, and fiber [9,22,23,24,25,26,27,28,29]. Dry milling uses different types of mills, air classifiers, and electrostatic separators to break down and fractionate grains or pulses into flours and ingredients [9,22], while wet milling applies wet extraction methods to separate protein, starch, and fiber from grains [24,26,28,29]. Fiber can be obtained in soluble and insoluble fractions. This paper focuses on the milling and fractionation of wheat and peas as these are common fractionation crops.

**Figure 1:**
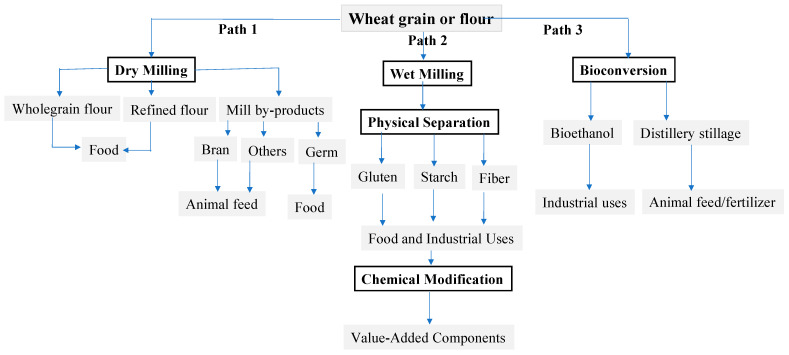
Current practices of wheat grain processing, including three production paths, roller milling, wet milling, and starch bioconversion.

Wheat gluten has a distinctive viscoelastic network structure that retains gas and holds food ingredients altogether in baked and other food products. This unique structure creates massive demand for gluten in the food and non-food industries. Gluten is a byproduct of the isolation of starch from wheat flour and has become a commodity due to its wide uses around the globe [30]. The extraction of gluten from wheat is an old process and is carried out by washing out starch and water-soluble components from wheat flour [31]. This method is still the basis of modern isolation processes of gluten, such as the Martin process or the Batter process [29]. Once the gluten is separated, the wet gluten requires a special drying method to preserve its techno-functionalities to produce vital gluten. Figure 1 shows the three main wheat fractionation industries, dry milling to make flour, wet milling to produce gluten, and bioconversion for ethanol production. Dry roller milling is the main process for making wheat flours, which are the principal ingredient in the baking industry. Wheat flour can be further processed into gluten and starch through wet milling. Four processes are employed by the industry in the separation of gluten and starch from wheat flour, including Martin, Alfa-Laval/Raisio, hydrocyclone, and high-pressure disintegration [26]. These processes primarily vary in the flour-to-water ratio and in the initial separation practice of starch and gluten fractions. The extraction of wheat starch and gluten can also be achieved from whole kernels, which often leads to impaired gluten due to the harsh processing conditions and/or interfering of bran soluble fiber components [29]. Figure 1 also shows the use of wheat grains in the production of bioethanol via a bioconversion process resulting in large qualities of distillery stillage byproduct. In this process, wheat starch is broken down into fermentable sugars by enzymes or acids, followed by yeast fermentation, to produce ethanol, which is recovered using a distillation process [32].

Corn is also an important fractionation crop in making starch, protein, and oil. It has a relatively large germ that is rich in oil, so corn grains are first degerminated to separate the germ for oil extraction prior to fractionation into starch and protein. The wet milling process is commonly used for the separation of corn kernels into their components through several steps, including soaking to soften grains, degermination to remove germ, and finally, the physical separation of starch and protein [33]. For the production of ethanol from corn, however, dry milling is preferred over wet milling because it requires less equipment, water, and capital costs [34,35]. Other cereal grains that are rich in health-enhancing components, such as arabinoxylan or β-glucan, could require special separation technologies. For instance, electrostatic separation has been found to selectively remove protein and starch from wheat bran to produce arabinoxylan concentrate with 43% arabinoxylan content [36]. Electrostatic separation technology in combination with ultra-fine grinding was used to produce β-glucan enriched fractions from oat bran with β-glucan content of 42% or 48% after one or two successive electrostatic separation steps, respectively [37].

**Figure 2:**
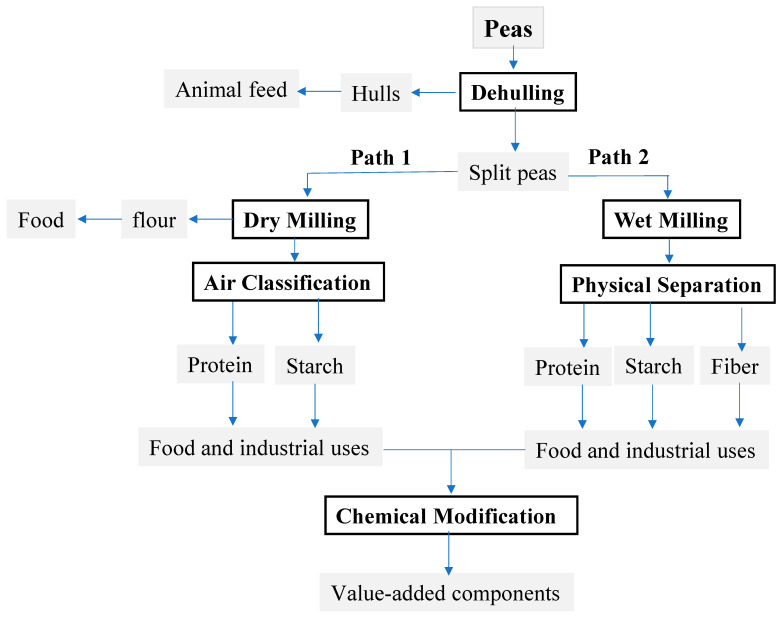
Current milling and fractionation of peas, including two processing paths, dry and wet milling.

Pulses are rich in proteins that are non-gluten; thus, there are increasing demands for their flours and proteins for making nutritious gluten-free food products. The gluten-free food market is rising at an annual growth rate of 7.6% from 2020 to 2027 [38]. In general, dry and wet milling are commonly used to fractionate pulses into protein and starch [28,39]. Figure 2 shows the dry fractionation (path 1) and wet fractionation (path 2) of peas into starch and protein from split peas after dehulling or hull removal. Dry fractionation is a chemical-free, no-water-use, and cost-effective process, but the protein purity is lower compared to that of the wet extraction method. In other words, the wet milling process produces protein isolates with a higher purity than that made by the dry fractionation process. For example, protein concentrates obtained from peas using dry milling and air classification exhibit protein contents of 51–55%, with a maximum protein recovery of 77% [25]. Nonetheless, protein isolates made from defatted peas by alkaline extraction and acid precipitation process contains 90% protein in comparison with 49% in the protein concentrate prepared using ethanol extraction [40]. Other pulses, such as beans and lupine, are also fractionated into high-protein ingredients for various uses and applications [41,42,43].

## 3. Limitations of Current Grain Milling and Fractionation Practices

Wheat and peas are common fractionation crops and can be considered appropriate examples for grain processing due to their popularity. The fractionation of wheat and peas involves a wide range of processing technologies, including primary processing (e.g., dehulling, milling, air classification, etc.) and value-added processes such as chemical modification and bioconversion (Figure 1 and Figure 2). A number of processes can also be set up in various layouts for making a variety of ingredients, subject to the purpose of the process. In general, the processing of grains and pulses produces a huge number of ingredients, ranging from refined or wholegrain flours, grain grits, and split seeds to single components such as starch, protein, and oil, in addition to a variety of value-added substances such as high-sugar syrups, ethanol, protein derivatives, modified starches, and others. These ingredients and products have been utilized in a wide range of applications, including food, cosmetics, paper, pharmaceutical, nutraceutical, healthcare, and others. The diversity of products and processes makes the milling and fractionation industry significantly important, but also more challenging and demanding. Current wheat milling and fractionation include three distinct processing paths, with a lack of coordination among them, as shown in Figure 1, while peas have two processing paths (Figure 2). This leads to the production of large amounts of byproducts, co-products, and waste. For example, in wheat roller milling, a lot of leftover parts of the wheat kernel are not added to the flour streams. These leftover parts (e.g., bran, shorts, and germ) are removed during the milling process and could be further processed into animal feeds and/or fertilizers or could be transformed into value-added ingredients, probably outside the milling facility. In the wet milling of wheat, corn, or peas into starch and protein, a large quantity of byproducts and waste are also produced and downgraded. In the brewing industry, a substantial quantity of byproducts is generated, primarily the brewer’s spent grains, which make up to 85% of brewing waste [44]. Most of these byproducts are downgraded and end up as animal feed or fertilizers [45]. The byproducts and waste issue is crucial in the grain industry, not only due to their negative impacts on the economy and the environment but also due to their contributions to food insecurity. The fragmentation and lack of coordination in wheat milling and fractionation also make the industry not cost-effective due to the cost of waste disposal and management of the supply chain. This problem is common among the fractionation of grains and pulses; in particular, the current design of processing facilities is based on a single purpose rather than multiple purposes, i.e., making flour, gluten, or ethanol in wheat processing.

In the wet milling industry, particularly the wet milling of corn, several studies have shown the excessive use of water, solvents, and energy [27,46]. For instance, the cost of energy in a typical corn wet milling plant in the USA is about $20–30 million per year [47]. Similarly, the isolation of starch from corn [48], arabinoxylan from wheat bran [36], or β-glucan from oat bran [37] consumes large amounts of water and requires an intensive drying process. Wet fractionation also uses rigorous conditions of temperature and pH, which could affect the techno- and bio-functionalities of the separated fractions. The low pH and high temperature employed in the extraction and isolation of protein modify its structure and functionality due to partial or full denaturation subject to the severity of processing conditions [27,49]. The high temperature of drying in the preparation of starch or during the processing of starchy foods affects its structural, pasting, and functional properties [50]. The wet extraction of arabinoxylan from wheat bran with an alkaline solution deteriorates its functionality by damaging its functional groups [36]. The application of heat in the preparation of β-glucan from cereals alters its structure, molecular weight, viscosity, and subsequently, its physiological properties [51]. These studies indicate that mild processing conditions are required in the wet fractionation of grains to preserve the techno- and bio-functionality of grain components.

In summary, grain fractionation processing faces a number of issues, including food waste, large amounts of byproducts and co-products, and the excessive use of water and energy, in addition to the rigorous processing conditions in wet milling. These processing constraints have negative impacts on food security and the environment, which requires a new concept to make the grain processing industry more sustainable and economically sound. The proposed model should be capable of adopting circularity in the milling and fractionation of grains and pulses through integration among processes, the versatility of equipment, and coordination among manufacturing and facility settings. It is also equally important to incorporate the valorization of byproducts as an integral part of the industry, along with the use of novel technologies. A novel approach is needed not only to avoid the existing fragmentation but also to cut down the excessive use of resources while making quality ingredients.

## 4. Sustainable Grain Processing System

As demonstrated in the previous section, large amounts of products are lost and/or wasted during the processing of grains and pulses into flour and other ingredients due to the absence of coordination and integration among industries. For the grain industry to effectively manage losses, waste, and resources, a more sustainable and circular grain processing system should be developed and adopted. The FAO defines a sustainable food system as “a food system that delivers food security and nutrition for all in such a way that the economic, social and environmental bases to generate food security and nutrition for future generations are not compromised” [52]. In the FAO definition, three principles, including economic, social, and environmental sustainability, are taken into consideration to secure an acceptable socioeconomic status and a healthy environment. In line with the FAO definition, a sustainable grain processing system can be defined as a system that is capable of producing nutritious and safe grain products through an economically sound processing industry that is efficient in the usage of water and energy and generates minimal environmental footprint. The system should incorporate the three principles outlined by the FAO due to the significant role of the grain sector in providing ingredients and products for human consumption, in addition to several other uses. The grain industry also contributes to environmental pollution. A study reported that the amount of CO_2_ emissions in the lifecycle of wheat products is dependent upon the agriculture system, processing method, and size of the regional area; in addition, within flour production, the organic flour process produces 12% lower CO_2_ emissions than that of the conventional flour production [53]. Without a doubt, the grain processing sector can play significant roles in the prevention of global hunger if a new vision to make it more sustainable and environmentally responsible is adopted. In this section, a novel sustainable model for processing wheat and peas into flours, proteins, starches, and value-added components is envisioned and discussed.

**Figure 3:**
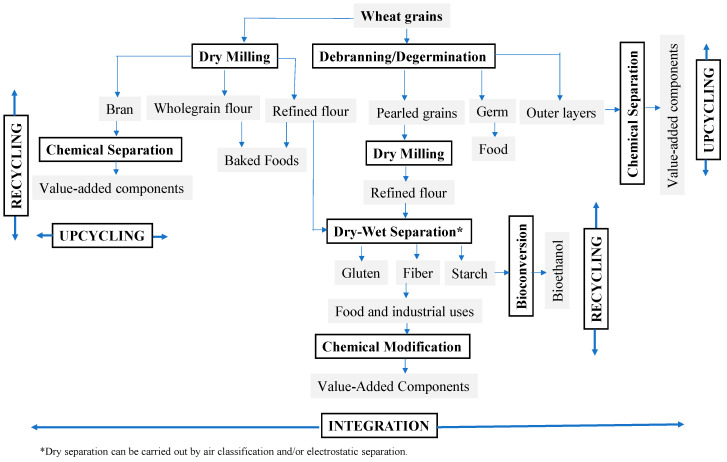
A proposed sustainable milling and fractionation system for wheat grains depicting the incorporation of the following three principles: integration, recycling, and upcycling.

**Figure 4:**
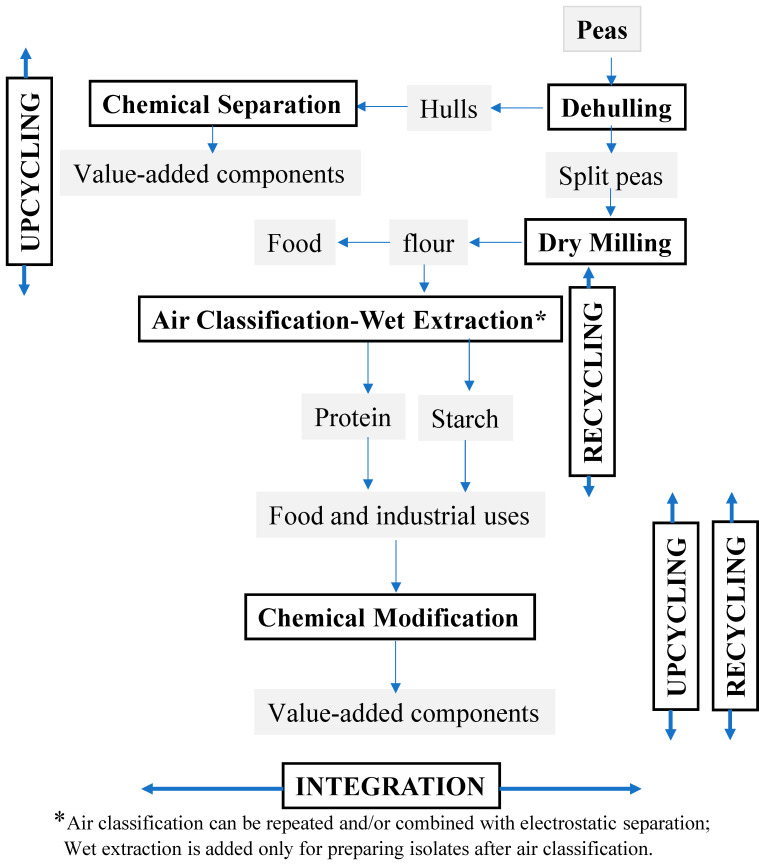
A proposed sustainable milling and fractionation system for peas depicting the incorporation of the three following principles: integration, recycling, and upcycling.

The proposed model for processing wheat (Figure 3) and peas (Figure 4) is based on three principles, namely, integration, recycling, and upcycling to implement circularity within the grain industry, with a potential for zero waste (e.g., <5% loss) through the efficient use of resources. In Europe, when the food system has been transformed toward circularity, it leads to sequential changes among its components, along with a great potential to preserve human and planetary health [54]. The European circular system is able to achieve a potential reduction of 71% in agricultural land use and 29% per capita in GHG emissions while still producing sufficient healthy foods. Other studies have shown that food waste and byproducts can be managed and valorized through a circular economic system [45,55]. In a circular economic system, food byproducts can safely be upcycled and reused for human consumption and/or industrial applications. The new model is based on the integration of the available milling and fractionation processing technologies via coordination to assist in diminishing the existing fragmentation. It also incorporates the recycling of water and component fractions to minimize waste and the excessive use of water and energy. Additionally, it employs the upcycling of waste and byproducts to transform them into value-added components via valorization technologies. The incorporation of the three principles should support the implementation of circularity in the industry.

Figure 3 presents a proposed hybrid model for a sustainable milling and fractionation system designed for wheat grains. The new model is based on a hybridization between dry and wet milling processes to facilitate the integration of the two processing methods and effectively manage resources. Studies have shown that wet milling is obsolete and uneconomical, with intensive uses of water and energy [34,47]. On the other hand, dry milling has shown great potential as a sustainable system for the separation of protein, starch, fiber, and other ingredients [9,22,27]. In this respect, a hybrid model would improve the efficiency of wheat milling and fractionation by minimizing waste and reducing water consumption, in addition to improving the quality of fractions and ingredients. The proposed model also incorporates de-branning (partial or full removal of the outer layers of kernels) as a pre-processing treatment prior to the separation of gluten and starch to enhance separation efficiency and component functionality while minimizing waste. Research has demonstrated that the de-branning of wheat results in improved flour quality and functionality [56]. The de-branning or pearling of barley enhances the cooking and nutritional quality of pot and pearl barley [57]. The separation sequence of protein, starch, and fiber from hairless canary seeds also affects the extraction efficiency and purity of the separated components, with protein and fiber fractions being more influenced by the order of extraction process compared to the starch fraction [58]. In general, the incorporation of de-branning would improve the separation efficiency of protein and starch and their techno-functionalities, as well. It will also enhance the processing efficiency of ethanol production from wheat starch by minimizing the amount of distillery stillage waste due to the removal of bran constituents.

Wheat is a versatile ingredient with several functionalities and applications for use in food and non-food products. It is also a good source of starch, protein, and dietary fiber, averaging 60, 17, and 13%, respectively. Thus, it is significantly beneficial for the global economy and the environment to transform wheat processing into a more sustainable industry. The proposed model shows two paths for wheat processing; the first path is required to produce refined and wholegrain flours for the baking industry, while the second path is a hybrid of the dry and wet milling systems to process pearled wheat flour into vital gluten and starch (Figure 3). The dry fractionation of pearled wheat can be performed using air classification and/or electrostatic separation for making protein and starch fractions, followed by chemical modification into value-added products. In addition, the proposed model makes a coordination to link the existing roller milling industry (path 1) with the hybrid system (path 2) to improve circularity within the system. The hybrid dry and wet milling system would reduce the amount of water/solvents consumed by the wet milling process due to the utilization of protein or starch fraction rather than the use of wholegrain or refined flour. The recycling of water in the system should also result in more water reduction. The new model also recommends merging the existing roller milling into the wet or dry fractionation to implement a new hybrid model and link to two processing paths. The goal is to eventually shift the existing wheat milling and fractionation into a coherent integrated and sustainable industry, which should support circularity and the efficient use of resources.

Corn is another important fractionation crop, but it is processed in a different way than wheat due to the differences in their kernel characteristics, morphology, and nutrient composition. The germ of corn is rich in oil, so the kernels are first degerminated to remove the germ for oil extraction, then the de-germinated grains are broken down into flours for fractionating it into starch and protein using physical separation methods. The inclusion of recycling and upcycling in corn wet milling would make it more sustainable and economically sound. A study has demonstrated the possibility to upcycle corn distillers’ dried grains with solubles and corncob into a balanced nutritional pig feed using co-fermentation with *Aspergillus niger* and *Trichoderma reesei* [59]. In corn wet milling, starch and oil are the primary products, while ethanol is produced from corn through dry milling, along with large quantities of byproducts, such as gluten meal and dried distillers’ grains with solubles, which are sources of valuable compounds. Corn byproducts can also be upcycled into several compounds, such as lignin, protein, carotenoids, and phenolic compounds, for food and industrial uses [60].

Upcycling is the reuse of food waste or byproducts in which, rather than being discarded, they are transformed into edible food ingredients and/or value-added substances. It holds promise for the grain processing industry to transform more byproducts and waste into valuable ingredients. It also helps shift the grain processing industry to be more sustainable by valorizing waste and reducing GHG emissions and environmental footprints [61]. Upcycling processes could be implemented onsite or offsite, preferably in close proximity to avoid changes in the composition of byproducts/waste and to make them more cost-effective. Cereal byproducts, such as bran, distiller’s grains, and brewer’s spent grains, are available in large quantities and can be exploited via upcycling processes. In other words, they can be converted into nutritionally valuable fractions, such as hemicelluloses (e.g., β-glucan and arabinoxylans) or proteins for food applications, whereas the insoluble fiber fractions can be used as feedstocks for bioethanol or the bulk chemical industry [62,63,64]. Brewer’s spent grains are also considered potentially promising materials for upcycling into value-added ingredients rich in protein, fiber, and phenolic compounds with a low environmental footprint [65,66].

Several bioprocessing methods and emerging technologies could be employed to implement upcycling in grain processing. The type of bioprocessing or processing technology is based on the type of byproduct or waste and potential end uses. A number of bioconversion methods using either enzymes or microorganisms, along with other technologies such as microencapsulation, supercritical extraction, microwave-assisted extraction, ultrasound, and pulse electric field, could be utilized for efficient recovery and product quality. Details about the valorization of byproducts and waste from cereal processing and plant-derived food waste into valuable ingredients and products have been previously discussed in recent review articles [45,55].

The size of the upcycled food market was valued at approximately $53.7 billion in 2021 and is estimated to reach $97 billion by 2031 [67]. Because upcycled foods are modified forms of ingredients that would be discarded, it is critical to assess them in terms of their sensory and nutritional attributes. One of the concerns is consumers’ acceptance and their willingness to purchase upcycled foods. According to a study on the reception of upcycled foods, there was good acceptance among consumers who are influenced more by a rational message rather than by an emotional one [68]. In another study, British consumers prefer biscuits made from wheat flour over biscuits containing upcycled sunflower ingredients based on four criteria, including price (high vs. low), type of flour (wheat vs. upcycled sunflower), protein sources (known vs. unknown), and carbon trust label (label vs. no label) [69]. In the latter study, the majority of participants showed a lack of awareness about the upcycled ingredients, but they would consider buying foods made from these ingredients. Overall, there is a need for more research on upcycled ingredients and foods to generate comprehensive data on their nutritional and sensory properties, as well as their impacts on the environment and food security. This knowledge is crucial for grain processors, consumers, and policymakers.

The application of artificial intelligence (AI) could also offer a great opportunity to make grain processing more effective and efficient. A recent review has emphasized four areas for the role of AI in wheat milling, including the prediction of flour quality and yield, energy saving, resource optimization, and mill performance [70]. In addition, AI methods have also been applied in the prediction of the quality of milled rice grains using a combination of near-infrared spectroscopy technology and machine learning algorithm models [71] or a combination of image processing and machine learning algorithm models to predict head rice yield [72]. Surely, machine learning models could provide a computational intelligence tool that can optimize milling and fractionation processes in terms of process optimization, water and energy saving, and product quality, but more research is needed in this area.

Dehulling, or the removal of hulls, is a crucial step in milling pulses, while splitting is carried out to split the seeds or cotyledons. Dehulling should be performed to fully remove the hull with a minimum amount of powder and broken seeds to improve dehulling yields and, subsequently, milling performance [73]. A proposed sustainable processing system for the fractionation of split peas into flour, protein, and starch, along with other value-added components, is presented in Figure 4. Due to the high contents of protein (20%) and starch (45%) in peas, the proposed model integrates dry fractionation processes, such as air classification, electrostatic separation, and other methods, to fractionate pea flour into protein and starch with high purity. The wet extraction process could also be merged into the system to further isolate protein and starch with higher purity and functionality than their corresponding concentrates. Recycling is also incorporated in the fractionation model for recycling either fractions in dry fractionation or water in the wet extraction process. Recycling protein or starch fraction can be performed by having two or more successive runs of air classification or electrostatic separation, or a combination of both processes, to produce more concentrated fractions of protein and starch compared to a single run of air classification or electrostatic separation. A combination of air classification and electrostatic separation technologies has been reported to produce highly concentrated protein and starch fractions compared to a single process [22]. Several studies have employed dry milling in combination with electrostatic separation to make protein-enriched fractions from navy bean flour [42] or lupine flour [43]. It has also been employed to produce β-glucan-enriched ingredients from oat bran [37] and arabinoxylan concentrates from wheat bran [36]. Dry milling followed by air classification has also been applied in making protein fractions from barley for food applications [74]. In another study, a hybrid method of dry and aqueous fractionation was used to produce protein fractions from quinoa with minimal impact on functionality [75]. Additionally, the use of the pre- and post-treatment of pulses, such as tempering, defatting, soaking, or freezing cycles, can also be applied to improve the purity of protein fractions obtained from pulses through dry milling and air classification [9,25]. In general, these studies have emphasized the benefits of dry fractionation in making protein or starch fractions with high purity and improved functionality and nutritional properties without the use of organic solvents. Upcycling co-products is also incorporated into the model to augment sustainability via value addition and the enhancement of byproducts. In general, the proposed model should be designed in a way that mimics a natural system for the efficient use of resources through integration, recycling, and upcycling to potentially produce zero waste. Surely, research is needed to assess the proposed model in terms of feasibility, effectiveness, and the quality of ingredients for wheat, peas, and other common fractionation crops.

**Figure 5:**
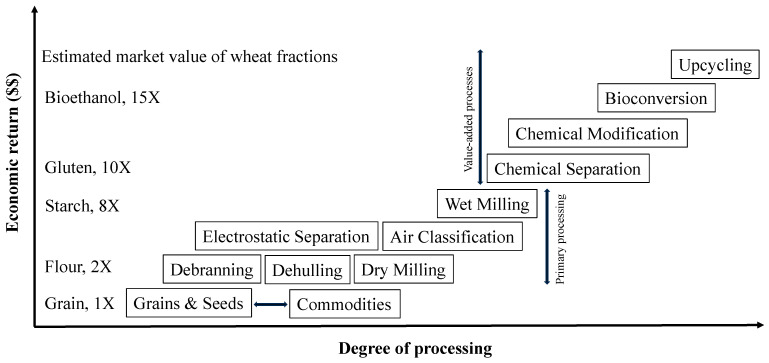
Impact of primary and secondary processing of grains on the economic return, along with the estimated market values of wheat fractions.

Lastly, the incorporation of value-added processes and upcycling as an integral part of crop fractionation should expand the utilization of fractions, co-products, byproducts, and waste. This should result in more value-added ingredients, along with improved sustainability and profitability. Figure 5 shows the impact of value-added processes on the economic returns of grain products by showing wheat products as examples. It shows the higher degree of value-added processing in product development, resulting in higher economic returns. For example, the estimated market values of ethanol and gluten made from wheat is about 10- and 15-fold compared to wheat grains, respectively (Figure 5). The further modification and/or separation of gluten or starch would result in valuable components with higher profits. The proposed model is designed to be an integrated system capable of making the mainstream flours, proteins, and starches, in addition to value-added products to boost economic values. In general, a good grain processing system should transform grains into value-added ingredients with zero waste to improve cost-effectiveness and economic return.

## 5. Conclusions

As the world population continues to grow, sustainable food systems are urgently needed more than ever to combat world hunger. They are also necessary to cope with the current issues that the global food production system faces, such as climate change, food loss and waste, and greenhouse gas emissions. Currently, most of the milling byproducts are downgraded and end up as animal feeds or fertilizers, which contributes to food insecurity and the environmental footprint. In this regard, a hybrid model for sustainable grain processing that combines dry and wet fractionation methods and incorporates three principles, including integration, recycling, and upcycling, is proposed and discussed. The new model is designed for sustainable milling and the fractionation of wheat and peas to enhance performance efficiency and economic return, along with a minimal environmental footprint. The model could boost food security through the efficient use of resources and the implementation of a circular economy. Surely, research is needed to assess the proposed model for the fractionation of wheat, peas, and corn in terms of the functionality of ingredients, performance efficiency, and material and energy balance. In addition, research on upcycled ingredients and foods is essential to provide processors, consumers, and policymakers with more information regarding their nutritional attributes and impacts on the environment and food security. Furthermore, machine learning models could provide a computational intelligence tool to assist the industry in milling process optimization, water and energy saving, and the prediction of product quality, but more research is needed in this area. Overall, this review provides potential solutions on how to make the grain processing system more sustainable.

## Data Availability

No new data were created or analyzed in this study. Data sharing is not applicable to this article.
